# Coxsackievirus and adenovirus receptor mediates the responses of endothelial cells to fluid shear stress

**DOI:** 10.1038/s12276-019-0347-7

**Published:** 2019-11-27

**Authors:** Jihwa Chung, Kyoung Hwa Kim, Shung Hyun An, Sunmi Lee, Byung-Kwan Lim, Sang Won Kang, Kihwan Kwon

**Affiliations:** 1 Medical Research Institute, School of Medicine, Ewha Womans University, Seoul, 07985 Republic of Korea; 2 Department of Life Science, College of Natural Science, Ewha Womans University, Seoul, 03760 Republic of Korea; 3 Department of Biomedical Science, Jungwon University, Goesan-gun, Chungbuk 28024 Republic of Korea; 4 Department of Internal Medicine, Cardiology Division, School of medicine, Ewha Womans University, Seoul, 07985 Republic of Korea

**Keywords:** Cell signalling, Atherosclerosis

## Abstract

Endothelial mechanotransduction by fluid shear stress (FSS) modulates endothelial function and vascular pathophysiology through mechanosensors on the cell membrane. The coxsackievirus and adenovirus receptor (CAR) is not only a viral receptor but also a component of tight junctions and plays an important role in tissue homeostasis. Here, we demonstrate the expression, regulatory mechanism, and role of CAR in vascular endothelial cells (ECs) under FSS conditions. Disturbed flow increased, whereas unidirectional laminar shear stress (LSS) decreased, CAR expression in ECs through the Krüppel-like factor 2 (KLF2)/activator protein 1 (AP-1) axis. Deletion of CAR reduced the expression of proinflammatory genes and endothelial inflammation induced by disturbed flow via the suppression of NF-κB activation. Consistently, disturbed flow-induced atherosclerosis was reduced in EC-specific CAR KO mice. CAR was found to be involved in endothelial mechanotransduction through the regulation of platelet endothelial cell adhesion molecule 1 (PECAM-1) phosphorylation. Our results demonstrate that endothelial CAR is regulated by FSS and that this regulated CAR acts as an important modulator of endothelial mechanotransduction by FSS.

## Vascular disease: Blood flow disturbances and atherosclerosis

Research into the mechanisms by which blood flow disturbances affect the function of endothelial cells (ECs), the cells lining the interior of blood vessels, reveals potential new targets for treating atherosclerosis. Kihwan Kwon at Ewha Womans University in Seoul, South Korea, and colleagues found that a membrane protein, the coxsackie and adenovirus *receptor*, *CAR*, mediates the response of ECs to the shear stress exerted by blood flow. They showed, in human tissue and in mice, that CAR protein levels in ECs increase when they are exposed to low or oscillatory blood flow, which is linked to the build-up of plaque inside arteries. Lowering CAR levels in ECs reduced the expression of proinflammatory genes and the formation of atherosclerotic lesions in mice. These findings suggest that reducing CAR activity could be a promising approach for treating atherosclerosis.

## Introduction

Endothelial cells (ECs) are constantly exposed to fluid shear stress (FSS) by blood flow. Changes in the pattern of FSS induce distinct cellular responses^1^. Disturbed flow, characterized by oscillatory and low shear stress, is associated with vascular inflammation and localization of atherosclerotic lesions, whereas laminar shear stress (LSS), characterized by a high unidirectional FSS force, is anti-inflammatory and atheroprotective^2^. Shear stress regulates EC function by stimulating a mechanosensory complex consisting of PECAM-1, vascular endothelial cadherin (VE-cadherin), and vascular endothelial growth factor receptor 2 (VEGFR2), followed by rapid activation of Src, phosphoinositide 3-kinase (PI3K), protein kinase B (Akt), and endothelial nitric oxide synthase (eNOS)^3^. In addition, several endothelial proteins or structures, including cell–cell junction molecules, integrins, ion channels, and the cytoskeleton, are involved in mechanotransduction^4^. This mechanotransduction signaling is required for various endothelial functions, such as proliferation, angiogenesis, migration, vasodilation, and inflammation.

The coxsackievirus and adenovirus receptor (CAR) is a viral receptor for group B coxsackieviruses and subgroup C adenoviruses^5,6^ and a component of intercalated discs of cardiomyocytes, epithelial tight junctions, and adherens junctions. Increased CAR expression in the myocardium is associated with cardiomyopathy^7^. In tumors, CAR exhibits tumor-suppressive activity by regulating cancer cell adhesion, proliferation, migration, and invasion^8–12^. In ECs, inflammatory cytokines, such as tumor necrosis factor alpha (TNF-α) and interferon gamma (IFN-γ), downregulate CAR expression^13^, and CAR mediates the transendothelial migration of neutrophils^14^. Although there is considerable knowledge of the function of CAR as a viral receptor, the expression patterns and physiological role of CAR in ECs under shear stress remain unclear.

Here, we show that CAR expression in ECs is regulated by FSS and that CAR plays an important role in FSS-induced mechanotransduction in ECs. CAR expression was increased under disturbed flow and decreased under LSS conditions, and these effects were regulated through the Krüppel-like factor 2 (KLF2)/activator protein 1 (AP-1) axis. Moreover, CAR was found to be associated with the mechanosensory complex under disturbed flow and modulate endothelial mechanotransduction signaling by regulating PECAM-1 phosphorylation. Inhibition of CAR expression decreased the expression of proinflammatory genes and endothelial inflammation, which were increased under disturbed flow, and disturbed flow-induced atherosclerosis was reduced in EC-specific CAR knockout (KO) mice. Our findings suggest that CAR plays an important role in FSS-induced mechanotransduction and may thus be a promising therapeutic target for disturbed flow-induced vascular diseases, such as atherosclerosis.

## Materials and methods

### Antibodies and reagents

Rabbit anti-CAR (sc-15405), mouse anti-CAR (sc-373791), rabbit anti-GAPDH (sc-25778), mouse anti-PECAM-1 (sc-365804), mouse anti-VE-cadherin (sc-9989), and phycoerythrin (PE)-conjugated anti-PECAM-1 (10G9) (sc-13537 PE) antibodies and phalloidin conjugate solution to stain F-actin were obtained from Santa Cruz Biotechnology, CA, USA). Rabbit anti-peNOS (S1177) (#9570), rabbit anti-pAkt (S473) (#4060), rabbit anti-pSrc (Y416) (#2113), rabbit anti-pSrc (Y527) (#2105), rabbit anti-VEGFR2 (#2479), rabbit anti-pIκBα (#2859), and rabbit anti-NF-κB p65 (#4764) antibodies were purchased from Cell Signaling Technology (Beverly, MA, USA). Mouse anti-phosphotyrosine (clone 4G10, 05-321) antibody was purchased from EMD Millipore (Billerica, MA, USA). Rabbit anti-VCAM-1 (ab134047), anti-PECAM-1 (ab28364), and anti-KLF2 (ab139699) antibodies were purchased from Abcam (Cambridge, UK). Rat anti-ICAM-1 (1701-01) antibody was purchased from Southern Biotech (Birmingham, AL, USA). Mouse anti-flag M2 antibody was purchased from Sigma-Aldrich (St. Louis, MO, USA). Rabbit anti-pPI3K p85 (Y458/Y199, P45-17387), Alexa Fluor 488-conjugated donkey anti-mouse IgG (A21202), and Alexa Fluor 568-conjugated donkey anti-rabbit IgG (A10042) antibodies were purchased from Thermo Fisher Scientific (Waltham, MA, USA). Rhodamine Red-X (RRX) goat anti-rat IgG (112-296-003) antibody was purchased from Jackson ImmunoResearch Laboratories (West Grove, PA, USA). A NF-κB inhibitor (PDTC; ammonium pyrrolidinedithiocarbamate), lipopolysaccharide (LPS; 1 μg/mL), an inducer of ER stress (thapsigargin [TG]; 5 μM), an inducer of oxidative stress (hydrogen peroxide [H_2_O_2_]; 0.1 mM), and Duolink in situ fluorescence reagents were purchased from Sigma-Aldrich. An AP-1 inhibitor (SR11302, [*E*,*E*,*Z*,*E*]-3-methyl-7-[4-methylphenyl]-9-[2,6,6-trimethyl-1-cyclohexen-1-yl]-2,4,6,8-nonatetraenoic acid) was purchased from Tocris Bioscience (Bristol, UK). Recombinant human TNF-α (20 ng/mL) protein was purchased from R&D Systems (Minneapolis, MN, USA). A human CD31 microbead kit was purchased from Miltenyi Biotec (Bergisch Gladbach, Germany).

### Cell culture

Primary human umbilical vein endothelial cells (HUVECs)^15^ and human vascular smooth muscle cells (HVSMCs)^16^ were isolated as described previously. We isolated HUVECs using a magnetic cell sorting (MACS) system and a human CD31 microbead kit (Miltenyi Biotec), and assessed their purity by measuring PECAM-1 expression using a FACScan flow cytometer (Becton Dickinson). HUVECs were cultured in Medium 200 (Invitrogen, Carlsbad, CA, USA)) with 5% fetal bovine serum (FBS; HyClone, Logan, UT, USA) and low-serum growth supplement (Invitrogen). HVSMCs were cultured in Dulbecco’s modified Eagle’s medium (DMEM; HyClone) with 10% FBS and a 1% antibiotic/antimycotic solution (Corning Cellgro, Manassas, VA, USA). Human U937 monocytes were purchased from the Korean Cell Line Bank (Seoul, Korea) and cultured in RPMI-1640 (HyClone) with 10% FBS and a 1% antibiotic/antimycotic solution. Cells were maintained at 37 °C in a humidified atmosphere containing 5% CO_2_.

### Fluid shear stress

Confluent HUVECs cultured in 60 -mm dishes were exposed to FSS in a cone-and-plate viscometer. Shear stress is specified in dyne per cm^2^, in which 1 dyne per cm^2^ is 0.1 Pa or 0.1 N per m^2^. We used a unidirectional steady flow (shear stress of 20 dyne/cm^2^) for LSS and a bidirectional disturbed flow (shear stress of ±5 dyne/cm^2^) for oscillatory shear stress (OSS), as described previously^17,18^. In the crossover experiment between LSS and OSS, HUVECs were exposed to LSS or OSS for 24 h, followed by exposure to the other type of shear stress for 24 h.

### Preparation of conditioned media

Conditioned media were collected from HUVECs under static or FSS conditions for 24 h and centrifuged at 3000× *g* for 10 min to remove cellular debris. To precipitate proteins from the conditioned media, the trichloroacetic acid (TCA) protein precipitation method was used. Briefly, 250 μL of chilled 100% TCA was added per 1 mL of conditioned media and incubated for 4 h at 4 °C. Then, the media were pelleted at high speed in a microcentrifuge at 4 °C, followed by two washes with ice-cold acetone. After the acetone was evaporated, protein pellets were solubilized in 2 × Laemmli sample buffer.

### Western blotting

HUVECs were harvested and lysed with radioimmunoprecipitation assay (RIPA) buffer (GenDEPOT, Barker, TX, USA) containing a 1% protease inhibitor mixture (GenDEPOT) and 1% phosphatase inhibitor (GenDEPOT). We measured the protein concentrations using a Pierce BCA protein assay kit (Thermo Fisher Scientific). Equal amounts of protein were subjected to sodium dodecyl sulfate–polyacrylamide gel electrophoresis (SDS-PAGE) and transferred to polyvinylidene difluoride (PVDF) membranes (GE Healthcare). Following incubation in a 5% skim milk solution prepared in 1 × Tris-buffered saline + Tween 20 for 1 h, membranes were probed with antibodies against CAR (1:1000; Santa Cruz Biotechnology), KLF2 (1:1000; Abcam), c-Jun (1:1000; Cell Signaling Technology), c-Fos (1:1000; Cell Signaling Technology), phospho-IκB (1:1000; Cell Signaling Technology), NF-κB p65 (1:1000; Cell Signaling Technology), phospho-PI3K (1:1000; Thermo Fisher Scientific), phospho-eNOS (1:1000; Cell Signaling Technology), phospho-Akt (1:1000; Cell Signaling Technology), phospho-Src pY416 (1:1000; Cell Signaling Technology), phospho-Src pY527 (1:1000; Cell Signaling Technology), PECAM-1 (1:1000; Santa Cruz Biotechnology), VE-cadherin (1:1000; Santa Cruz Biotechnology), VEGFR2 (1:1000; Cell Signaling Technology), phosphotyrosine clone 4G10 (1:1000; EMD Millipore), flag M2 (1:1000, Sigma-Aldrich), GAPDH (1:1000; Santa Cruz Biotechnology), and Lamin A/C (1:1000; Santa Cruz Biotechnology). The total protein levels were normalized to the levels of GAPDH or Lamin A/C to control for loading. Finally, the membranes were stripped and reprobed with anti-GAPDH antibody.

### Transfection with siRNA and plasmids

To knockdown KLF2, c-Jun, c-Fos, and CAR, double-stranded siRNA and a scrambled siRNA control were purchased from Bioneer. Transfection with 50 nM siRNA was performed using Lipofectamine RNAiMAX (Invitrogen) in OptiMEM (Invitrogen) according to the manufacturer’s instructions. After transfection for 24 h, HUVECs were exposed to LSS for 24 h. To overexpress KLF2, components of the AP-1 complex (c-Fos and c-Jun), and CAR, we used the human KLF2 construct (PpyCAG-KLF2-IB), a gift from Austin Smith (Addgene plasmid #60441); the human c-Fos construct (pLX304-FOS-V5), a gift from William Hahn (Addgene plasmid #59140), and the human c-Jun construct (pMIEG3-c-Jun), a gift from Alexander Dent (Addgene plasmid #40348); and the flag-tagged human CAR construct, a gift from Prof. Lim (Jungwon University, Republic of Korea), respectively. Transient DNA transfection was performed using Lipofectamine 3000 (Invitrogen) in OptiMEM (Invitrogen) according to the manufacturer’s instructions. After 48 h of transfection with 5 μg of plasmid, HUVECs were exposed to LSS or OSS for the indicated periods of time. To validate the efficacy of KLF2, c-Jun, c-Fos, and CAR gene knockdown and overexpression in ECs, we determined the KLF2, c-Jun, c-Fos, or CAR protein levels by western blotting.

### Luciferase reporter assay

For the luciferase reporter assay, the following plasmids were used: firefly luciferase reporter plasmid for AP-1 (3xAP-1pGL3), a gift from Alexander Dent (Addgene plasmid #40342); *Renilla* luciferase normalization construct (pRL-SV40P), a gift from Ron Prywes (Addgene plasmid #27163); and a plasmid-encoding KLF2 (PpyCAG-KLF2-IB), a gift from Austin Smith (Addgene plasmid #60441). HUVECs were cotransfected in a 60 -mm dish with 5 μg of reporter (3xAP-1pGL3), 0.5 μg of the normalization construct (pRL-SV40P), and 5 μg of plasmid-encoding KLF2 using Lipofectamine 3000 (Invitrogen). After 24 h of transfection, cells were exposed to LSS or OSS for 24 h and then lysed. We measured AP-1 promoter activity using a dual-luciferase reporter assay system (Promega) with a Sirius FB12 Single Tube Luminometer (Berthold Detection Systems) according to the manufacturer’s instructions.

### Nuclear/cytosolic fractionation

Nuclear and cytosolic cellular fractions were prepared using a Nuclear/Cytosol Fractionation Kit (BioVision, Milpitas, CA, USA) following the manufacturer’s instructions. All steps were performed on ice. The purity of the protein fractions was assessed by immunoblotting with the anti-Lamin A/C (nuclear protein) and anti-GAPDH (cytoplasmic protein) antibodies.

### Monocyte-binding assay

HUVECs were transfected with 50 nM siRNA targeting CAR using Lipofectamine RNAiMAX (Invitrogen). After 24 h of transfection, HUVECs were exposed to LSS or OSS for 24 h. Human U937 monocytes (5 × 10^5^) were added to the HUVECs and incubated for 1 h at 37 °C. Unbound cells were removed by washing in serum-free medium. Bound cells were counted in five randomly selected fields per well. Phase-contrast micrographs of cells were obtained using an Olympus CKX41 microscope.

### Animal model of disturbed flow-induced atherosclerosis

Mice with partial carotid artery ligation were generated as described previously^19^. Briefly, WT male C57BL/6 mice (7 weeks old; Central Lab Animal, Seoul, South Korea) and EC-specific CAR KO mice (7 weeks old) were anaesthetized by the intraperitoneal injection of a mixture of Zoletil (30 mg/kg) and Rompun (10 mg/kg). The left carotid artery (LCA) was exposed by blunt dissection. All branches of the left carotid arteries, including the left external carotid, internal carotid, and occipital arteries, but not the superior thyroid artery, were ligated. The incision was closed with TissueMend. Mice were monitored in a chamber on a heating pad after surgery. For quantitative reverse transcription polymerase chain reaction (qRT-PCR) and en face staining, WT and EC-specific CAR KO mice were fed a regular mouse chow diet until euthanization. For atherosclerosis studies, WT and EC-specific CAR KO mice were fed a western diet for 4 weeks.

### Isolation of endothelial cell-enriched RNA from carotid arteries

Mice were euthanized by CO_2_ inhalation and pressure perfused with saline containing heparin (10 U/mL) via the left ventricle after severing of the inferior vena cava. Next, we isolated and carefully cleaned periadventitial fat from the partially ligated LCA and unligated RCA as described previously^19,20^. Briefly, we flushed the lumen of both carotid arteries for a few seconds with 200 μL of QIAzol lysis reagent (Qiagen) using a 29-gauge insulin syringe in a microfuge tube. The eluent was used to isolate endothelial cell-enriched RNA with QIAzol (Qiagen) according to the manufacturer’s instructions.

### Reverse transcription (RT) PCR and real-time PCR

We extracted the total RNA from HUVECs using QIAzol reagent (Qiagen) according to the manufacturer’s instructions. We quantified the RNA concentration using a NanoDrop spectrophotometer (Thermo Fisher Scientific). We synthesized single-stranded complementary DNA (cDNA) using M-MLV reverse transcriptase (Promega) and oligo-dT 15 primer (Promega). cDNA was amplified by RT-PCR over 30 cycles using a Mastercycler (Eppendorf). Real-time PCR was performed with SYBR Green PCR Master Mix (Qiagen) on an ABI StepOne Real-Time PCR System (Applied Biosystems).

### En face staining

Mouse aortas were collected from WT C57BL/6 mice 3 days after ligation. Isolated aortas were fixed in 4% paraformaldehyde for 20 min at room temperature. After permeabilization in 0.05% Triton X-100 (in PBS) for 20 min and blocking in 10% donkey animal serum for 1 h at room temperature, the aortas were incubated with rabbit anti-CAR antibody (1:200; Santa Cruz Biotechnology) overnight at 4 °C. After washing in PBS three times, the aortas were incubated with Alexa Fluor 568-conjugated donkey anti-rabbit (1:400; Invitrogen) IgG secondary antibody for 2 h at room temperature. We detected the fluorescence signal using a Zeiss LSM 800 confocal microscope.

### Immunofluorescence staining

For the colocalization assay, HUVECs exposed to FSS were fixed in 4% paraformaldehyde for 20 min at room temperature and permeabilized in 0.05% Triton X-100 (in PBS) for 20 min. After blocking in 10% donkey serum for 1 h at room temperature, cells were incubated with mouse anti-CAR (1:50) and rabbit anti-PECAM-1 (1:50) antibodies for 3 h at 37 °C. After washing in PBS three times, cells were incubated with Alexa Fluor 568-conjugated donkey anti-rabbit (1:400; Invitrogen) or Alexa Fluor 488-conjugated donkey anti-mouse IgG (1:400; Invitrogen) secondary antibodies for 2 h in the dark at room temperature. For the stress fiber-formation assay, HUVECs transfected with siRNA against CAR were exposed to FSS for 24 h, fixed in 4% paraformaldehyde for 20 min at room temperature, and permeabilized in 0.1% Triton X-100 (in PBS) for 5 min. After washing, cells were incubated with phalloidin conjugate solution to stain F-actin for 1 h in the dark at room temperature. For in vivo analysis, 3 days after carotid artery ligation surgery in WT and EC-specific CAR KO mice, the carotid arteries were isolated, embedded in optimum cutting temperature (OCT) medium, and frozen at –80 °C. Human normal control arteries (lobar pulmonary artery and internal mammary artery) were harvested from lung cancer patients who had undergone curative surgical resection, and atherosclerotic plaques (atheroma of anterior tibial artery) were harvested from patients who had undergone amputation of a lower extremity due to severe ischemic necrosis. Hematoxylin and eosin (H&E) was used for basic tissue morphological analysis. Immunofluorescence staining was performed as described previously^20^. Briefly, the sections were fixed in 4% paraformaldehyde for 20 min at room temperature and permeabilized in 0.05% Triton X-100 (in PBS) for 20 min. After blocking in 10% donkey serum for 1 h at room temperature, sections were incubated with rabbit anti-CAR (1:100), mouse anti-PECAM-1 (1:50), rabbit anti-eNOS (1:50), rabbit anti-VCAM-1 (1:200), or rat anti-ICAM-1 (1:500) antibody overnight at 4 °C. After washing in PBS three times, sections were incubated with Alexa Fluor 568-conjugated donkey anti-rabbit IgG (Invitrogen), Alexa Fluor 488-conjugated donkey anti-mouse IgG (Invitrogen), or Rhodamine Red-X (RRX) goat anti-rat IgG (Jackson ImmunoResearch Laboratories) secondary antibody in PBS for 2 h in the dark. Nuclei were counterstained with 4’,6-diamidino-2-phenylindole (DAPI; 100 ng/mL; Santa Cruz Biotechnology) for 5 min. The sections were mounted on glass coverslips with fluorescence mounting medium (DAKO, Glostrup, Denmark). Sections were visualized and analyzed using a Zeiss LSM 800 confocal microscope.

### Oil Red O staining of atherosclerotic plaques in mouse carotid arteries

Four weeks after ligation, carotid arteries were isolated from WT and EC-specific CAR KO mice, embedded in OCT medium, and frozen at –80 °C. Sections 7 μm in thickness were prepared. After fixation in 10% formalin for 5 min at room temperature, tissues were washed twice in water and then in propylene glycol for 5 min. Carotid arteries were stained with Oil Red O for 5 min at 65 °C, rinsed in 85% propylene glycol for 5 min, and rinsed three times in water. The samples were counterstained with hematoxylin and rinsed three times in water. After mounting, we visualized the samples using an Olympus BX51 microscope. We measured the IMT by calculating the luminal area relative to the total vascular area using National Institutes of Health ImageJ software.

### Quantification of PECAM-1 phosphorylation

All samples to be quantified from a set of experiments were handled as a unit for immunoprecipitation (IP), SDS-PAGE, and immunoblotting. HUVECs were transfected with 50 nM siRNA or 5 μg of plasmid-encoding CAR for 48 h and exposed to LSS or OSS for 10 min. The cells were then washed with ice-cold PBS and lysed with lysis buffer (50 mM Tris-HCl [pH 8.0], 5 mM EDTA, 150 mM NaCl, 1% NP-40, 1 mM PMSF, 2 mM Na_3_VO_4_, and 1% protease inhibitor cocktail). The lysate was centrifuged at 12,000 rpm for 20 min at 4 °C. Approximately, 500 μg of protein was used for IP. Cell lysates were first precleared with 2 μg of mouse IgG-agarose (Santa Cruz Biotechnology) for 1 h to eliminate nonspecific protein binding with IgG or agarose. The agarose beads were spun down at 2500 rpm for 5 min, and the supernatant was incubated with 2 μg of anti-PECAM-1 (Santa Cruz Biotechnology) for 16 h at 4 °C with continuous mixing. In the negative controls, mouse IgG was added as a substitute for primary antibodies. We precipitated the immunocomplexes by incubation with 20 μL of Protein A/G Plus-Agarose (Santa Cruz Biotechnology) for 3 h with constant agitation; then, the immunocomplexes were harvested by centrifugation. After washing three times in 400 μL of lysis buffer containing protease and phosphatase inhibitors with gentle resuspension and centrifugation (2500 rpm, 5 min), precipitated immunocomplexes were eluted by heating at 100 °C for 5 min in 1 × SDS sample buffer (Bio-Rad Laboratories), subjected to SDS-PAGE, and immunoblotted. To assess the phosphorylation of PECAM-1, we immunoblotted PECAM-1 immunoprecipitate with anti-phosphotyrosine (1:1000; EMD Millipore) and rabbit anti-PECAM-1 (1:1000; Abcam) antibodies. The level of PECAM-1 phosphorylation was determined as the ratio of phosphotyrosine to PECAM-1.

### Proximity ligation assay

We determined endogenous protein–protein interactions between CAR and the mechanosensory complex (VEGFR2, VE-cadherin, and PECAM-1) using Duolink in situ fluorescence reagents (Sigma-Aldrich). HUVECs adhered to coverslips were transfected with siRNA against CAR for 48 h or exposed to OSS for 24 h and fixed with 2% paraformaldehyde at room temperature for 20 min. After washing with PBS, cells were blocked with 5% normal donkey serum (in 0.5% Triton X-100) for 30 min. Cells were stained with anti-CAR (1:50; Santa Cruz Biotechnology), anti-VEGFR2 (1:50; Cell Signaling Technology), anti-VE-cadherin (1:50; Santa Cruz Biotechnology), or anti-PECAM-1 (1:50; Santa Cruz Biotechnology) antibody. After washing with 0.2% BSA in PBS, coverslips were stained with Duolink probes per the manufacturer’s instructions (Sigma-Aldrich), and probe-conjugated secondary antibodies were added and incubated for 1 h at 37 °C. Next, ligation was performed using the provided ligase for 30 min, followed by amplification using the provided polymerase for 100 min at 37 °C. Nuclei were counterstained with DAPI (100 ng/mL; Santa Cruz Biotechnology) for 5 min. We detected protein–protein interactions between CAR and the mechanosensory complex using a Zeiss LSM 880 Airyscan confocal microscope.

### Statistical analysis

Data are expressed as the means ± standard errors of the mean (SEMs) of at least three independent experiments. We tested quantitative variables using the nonparametric Mann–Whitney *U* test. Differences between groups were considered significant at *P* < 0.05.

## Results

### FSS regulates CAR expression in ECs

**Fig. 1:**
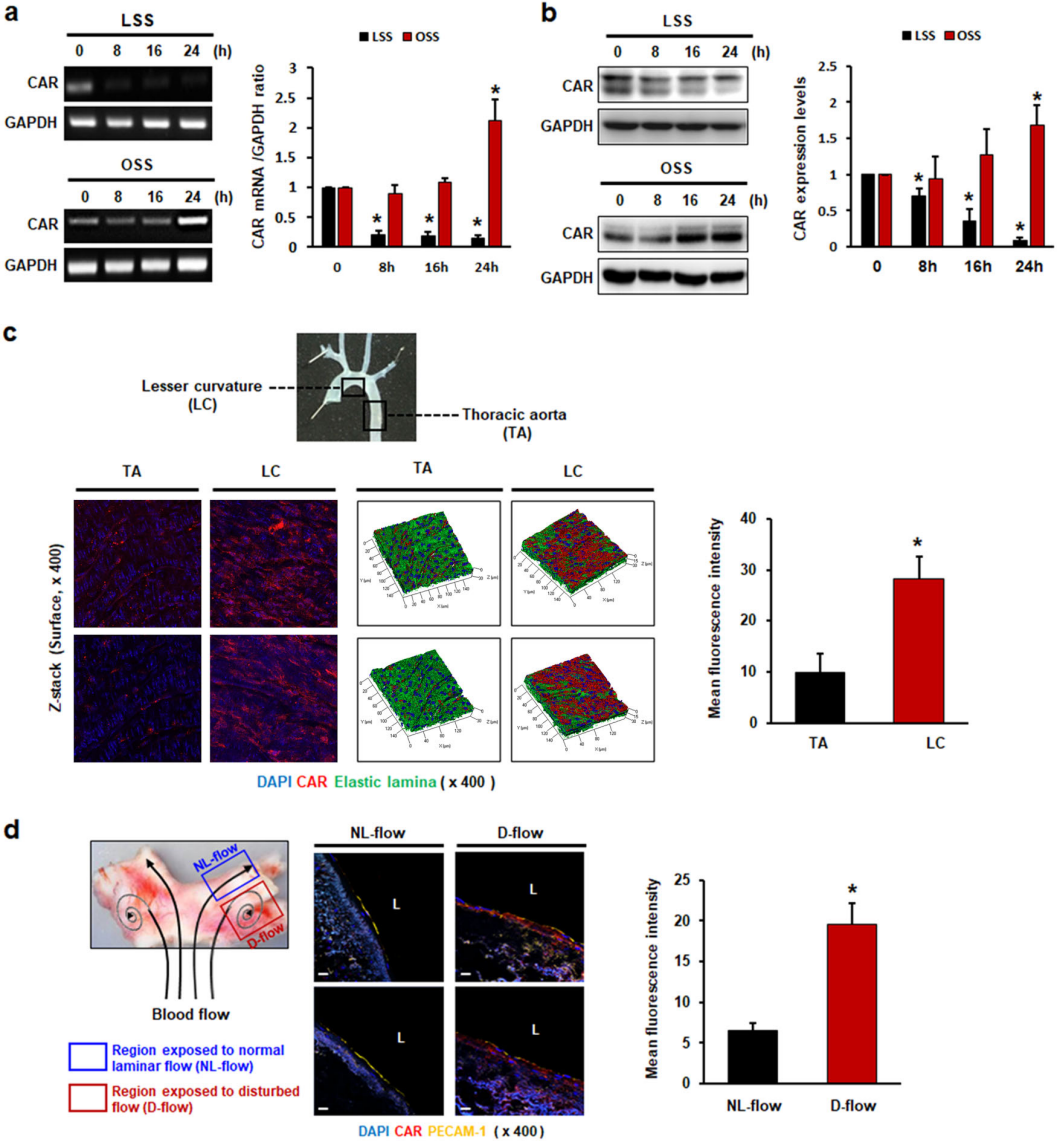
Fluid shear stress regulates CAR expression in endothelial cells. HUVECs were cultured under static, LSS, or OSS conditions for the indicated periods of time. **a** Levels of mRNAs encoding CAR and GAPDH (internal control) were determined by RT-PCR. **b** CAR protein levels were measured by western blotting. Representative images are shown (*n* *=* 5; compared with static conditions, *P *<* 0.05). **c** Mouse aortas were divided into two regions according to the distribution of vessel wall shear stress: the lesser curvature (LC; disturbed flow) and thoracic aorta (TA; normal laminar flow)^20^. The expression of CAR was evaluated by en face staining and confocal microscopy. Localization of CAR on the surface of the endothelium was verified by z-stack imaging (red, CAR; blue, nuclei; green, elastic lamina of vessel). **d** Frozen sections of human artery tissues^20^ were stained with anti-PECAM-1 and anti-CAR antibodies. PECAM-1 was used as an endothelial-specific marker. The expression of CAR was compared in two regions of the bifurcation site of the vessel with different wall shear stresses, the outer side with disturbed flow (D-Flow) and the inner side with normal laminar flow (NL-Flow), by confocal microscopy. Representative images from at least three experiments are shown (red, CAR; yellow, PECAM-1; blue, nuclei). Scale bars, 10 μm.

We first investigated whether FSS regulates endothelial CAR expression by measuring CAR expression in HUVECs subjected to LSS or disturbed flow. Interestingly, disturbed flow upregulated, whereas LSS downregulated, CAR expression at both the mRNA (Fig. 1a) and protein (Fig. 1b) levels. Detection of CAR protein by western blotting showed two bands, one at ~40 kDa and one at 46 kDa. The extracellular portion of CAR has a two-immunoglobulin (Ig)-like domain for N-glycosylation^21^. Peptide *N*-glycosidase F (PNGase F) reduced the size of the 46 kDa band to 40 kDa, suggesting that this shift in molecular size was due to deglycosylation^22^. We found that changes in the FSS pattern regulated mainly CAR expression, indicated by a band at 40 kDa. We also examined CAR expression following the transition from LSS to OSS and vice versa. Reduced CAR expression in LSS was recovered by subsequent OSS, whereas increased CAR expression in OSS was reduced by subsequent LSS (Supplementary Fig. 1). Recently, a wide variety of cell surface receptors have been shown to undergo proteolysis by the A Disintegrin and Metalloprotease (ADAM) family, the members of which cleave and release the ectodomains of cell surface proteins. CAR was found to undergo ectodomain shedding by the metalloprotease ADAM10 and regulated intramembrane proteolysis (RIP) in glioma cells^23^. Next, we explored whether decreased CAR expression in LSS was due to CAR ectodomain shedding. To investigate the possibility of CAR ectodomain shedding in ECs under FSS, we collected conditioned media from HUVECs exposed to FSS and performed western blot analyses using antibody against the extracellular domain. Full-length CAR with a molecular weight of ~46 kDa was detected in cell lysates. An extracellular fragment of CAR (~32 kDa) indicating ectodomain shedding was found in conditioned media, and its concentration was higher under FSS conditions than under static conditions. However, HUVECs exposed to OSS showed increased CAR ectodomain shedding compared with those exposed to LSS (Supplementary Fig. 2). These results suggest that the regulation of endothelial CAR expression by FSS is not due to shedding of the CAR ectodomain. In addition, we determined the level of CAR expression in HUVECs in response to various stimuli, including TNF-α. In contrast to a previous report^13^, the inflammatory cytokine TNF-α upregulated CAR expression in our study. Thapsigargin (TG), an inducer of endoplasmic reticulum (ER) stress, downregulated CAR expression, but lipopolysaccharide (LPS) and hydrogen peroxide (H_2_O_2_; an inducer of oxidative stress) had no effect on CAR expression (Supplementary Fig. 3). To investigate the effects of FSS on endothelial CAR expression in vivo, we assessed CAR expression in mouse aortic tissues naturally exposed to various blood flow patterns by en face staining. CAR expression was higher in the lesser curvature (LC) region, which is exposed to disturbed flow, than in the thoracic aorta (TA) area, which is exposed to LSS. CAR expression in the region of disturbed flow was predominantly localized on the luminal surface of the endothelium, as shown by z-stack surface imaging (Fig. 1c). Next, we investigated endothelial CAR expression in human arterial tissue. Consistent with mouse aortic tissue, the regions exposed to disturbed flow showed higher levels of CAR expression than regions exposed to normal laminar flow (Fig. 1d).

### CAR expression in ECs is regulated by the flow-sensitive KLF2/AP-1 axis

**Fig. 2:**
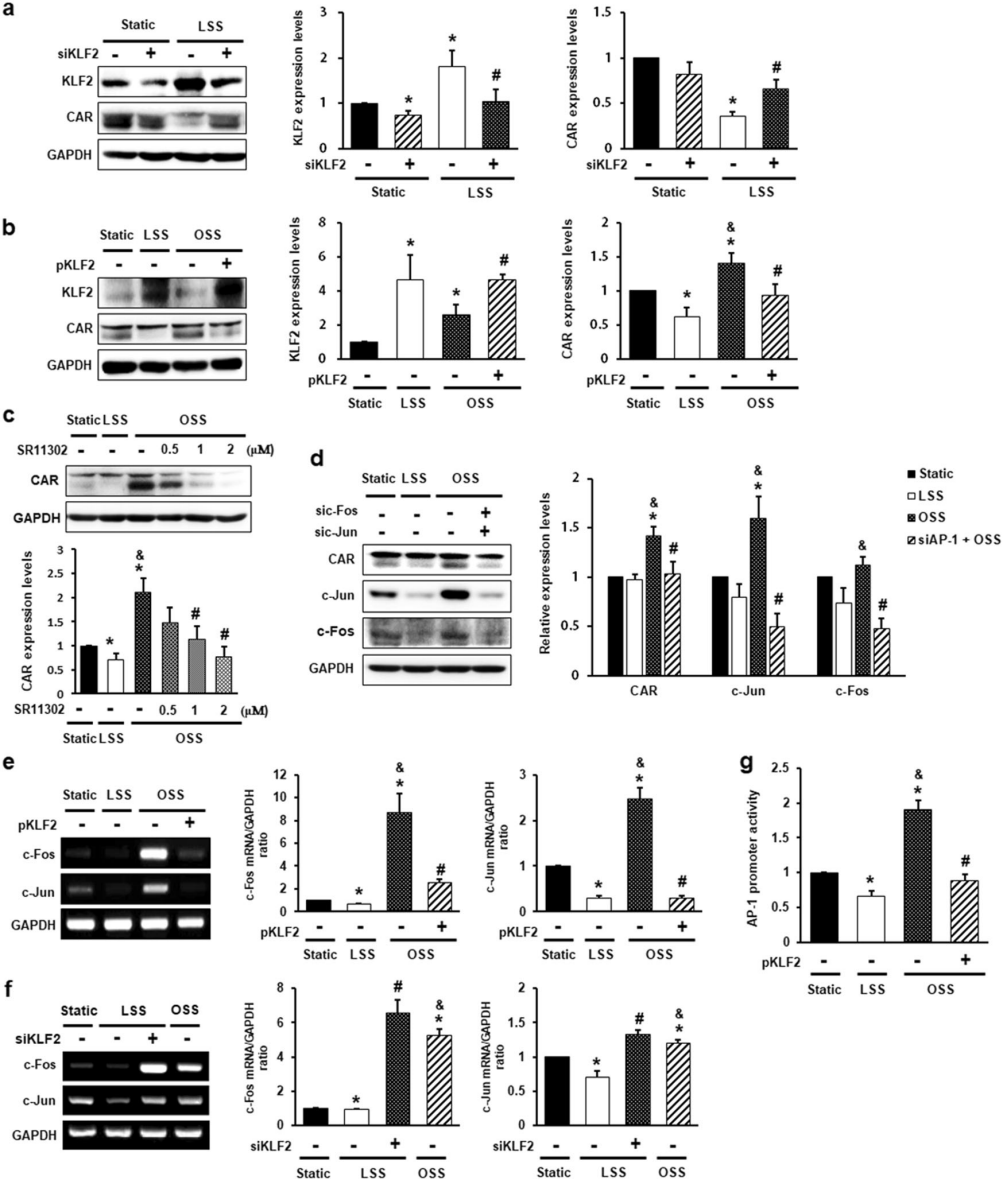
CAR expression in endothelial cells is regulated by the flow-responsive KLF2-AP-1 axis. HUVECs were transfected with siRNA against Krüppel-like factor 2 (KLF2; 50 nM) (**a**, **f**) or plasmid-encoding KLF2 (5 μg) (**b**, **e**) and then exposed to FSS for 4 h or 24 h. KLF2 and CAR protein levels were measured by western blotting. **a** *n* = 5; *P < 0.05, static vs. LSS; ^#^*P* < 0.05, LSS vs. siKLF2 + LSS. **b** *n* = 5; *P < 0.05, static vs. LSS or OSS; ^&^*P* < 0.05, LSS vs. OSS; ^#^*P* < 0.05, OSS vs. pKLF2 + OSS. **c** HUVECs were exposed to FSS for 24 h after pretreatment with the AP-1 inhibitor SR11302 for 1 h. CAR protein levels were detected by western blotting (*n* = 7; *P < 0.05, static vs. LSS or OSS; ^&^*P* < 0.05, LSS vs. OSS; ^#^*P* < 0.05, OSS vs. SR11302 + OSS). **d** HUVECs transfected with siRNAs against c-Fos (100 nM) and c-Jun (100 nM) were exposed to FSS for 24 h. The protein levels of CAR, c-Fos, and c-Jun were measured by western blotting (*n* = 5; *P < 0.05, static vs. LSS or OSS; ^&^*P* < 0.05, LSS vs. OSS; ^#^*P* < 0.05, OSS vs. siAP-1 + OSS). **e**, **f** Transfected HUVECs were exposed to FSS for 4 h. Levels of mRNAs encoding AP-1 components (c-Jun and c-Fos) and GAPDH were measured by RT-PCR. **e** *n* *=* 4; *P < 0.05, static vs. LSS or OSS; ^&^*P* *<* 0.05, LSS vs. OSS; ^#^*P* < 0.05, OSS vs. pKLF2 + OSS. **f** *n* *=* 4; *P < 0.05, static vs. LSS or OSS; ^&^*P* *<* 0.05, LSS vs. OSS; ^#^*P* < 0.05, LSS vs. siKLF2 + LSS. **g** AP-1 promoter activity was measured by luciferase reporter assay (*n* = 5; *P < 0.05, static vs. LSS or OSS; ^&^*P* < 0.05, LSS vs. OSS; ^#^*P* < 0.05, OSS vs. pKLF2 + OSS).

KLF2 is a flow-responsive key shear stress-induced transcription factor that governs the expression of many flow-regulated endothelial genes^24–26^. CAR expression was inversely correlated with KLF2 expression following shear stress (Fig. 2a, b). Therefore, we hypothesized that KLF2 regulates CAR expression in ECs. To test this hypothesis, we evaluated CAR expression in HUVECs transfected with siRNA against KLF2 or plasmids encoding KLF2. As expected, CAR expression, which is reduced under LSS, increased when KLF2 was silenced (Fig. 2a). In contrast, increased CAR expression under OSS decreased when KLF2 was overexpressed (Fig. 2b). In addition, KLF2 downregulated the expression of proinflammatory genes by inhibiting AP-1^26,27^ and nuclear factor kappa B (NF-κB) activation^28^. Interestingly, inhibition of NF-κB with the inhibitor pyrrolidinedithiocarbamate (PDTC) had no effect on CAR expression under disturbed flow (Supplementary Fig. 4). To investigate whether AP-1 is associated with CAR expression under flow conditions, we exposed HUVECs to shear stress after pretreatment with SR11302, an AP-1 inhibitor. AP-1 inhibition decreased CAR upregulation in OSS (Fig. 2c). Consistent with this finding, increased CAR expression under OSS decreased when c-Jun and c-Fos, components of the AP-1 complex, were silenced (Fig. 2d). Next, we examined whether the suppression of CAR by KLF2 is mediated by AP-1 activation blockade. To detect the effects of KLF2 on AP-1 activation, we evaluated c-Jun and c-Fos expression in HUVECs following the overexpression or knockdown of KLF2. KLF2 overexpression abolished the OSS-induced increase in the mRNA levels of c-Jun and c-Fos (Fig. 2e), whereas KLF2 knockdown cells under LSS conditions showed higher mRNA levels of c-Jun and c-Fos than those in cells under LSS conditions (Fig. 2f). OSS consistently enhanced AP-1 promoter activity compared with that under LSS. However, KLF2 overexpression suppressed the OSS-induced increase in AP-1 promoter activity (Fig. 2g). Therefore, the expression of CAR in ECs is regulated by the flow-responsive KLF2/AP-1 axis.

### CAR knockdown inhibits disturbed flow-induced inflammatory responses in ECs

**Fig. 3:**
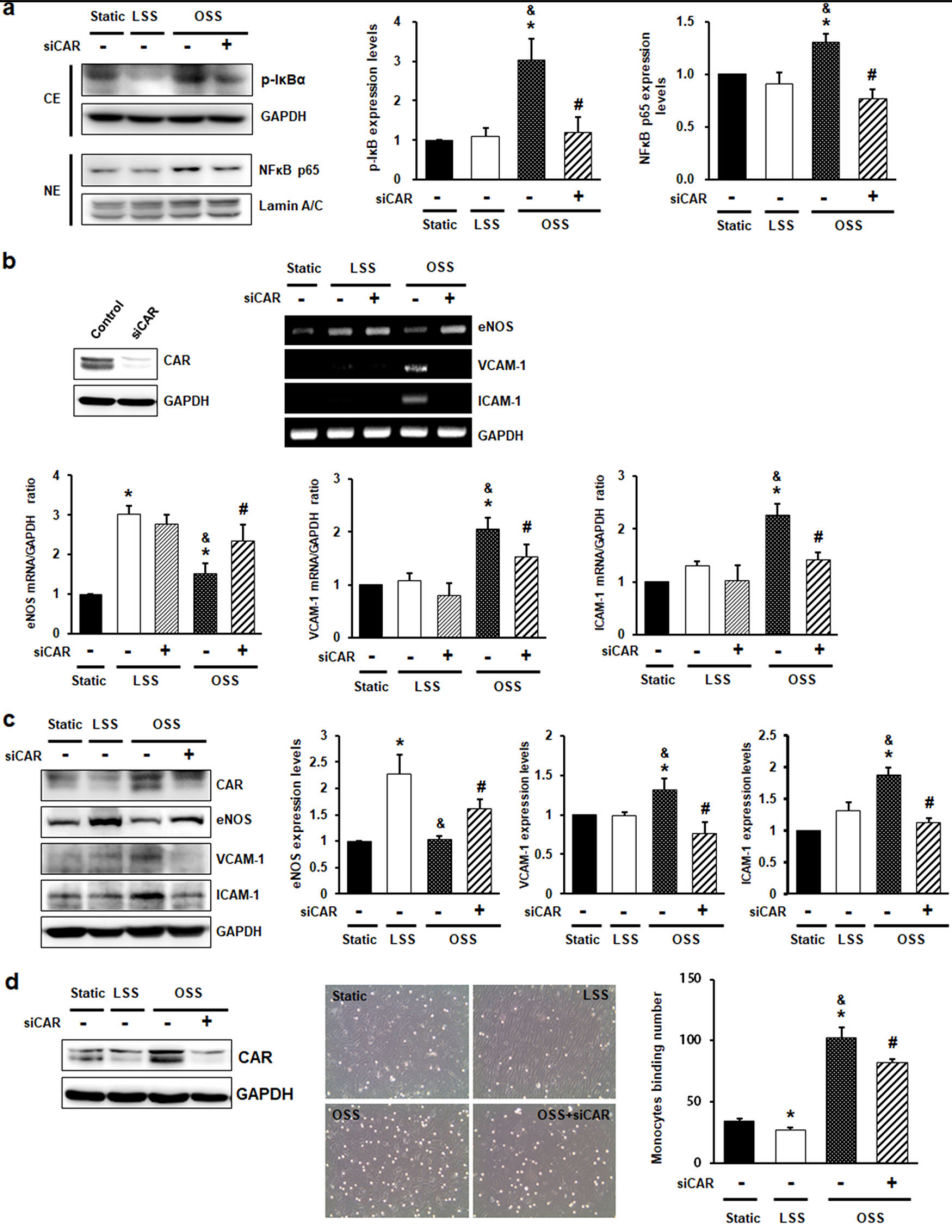
CAR knockdown inhibits disturbed flow-induced inflammatory responses in endothelial cells. HUVECs transfected with siRNA against CAR were exposed to FSS for 6 h (**b**) or 24 h (**a**). **a** Cytosolic and nuclear fractions were separated after 24 h of FSS. The extent of IκB phosphorylation and nuclear translocation of NF-κB were determined by western blotting with antibodies against p-IκB, NF-κB p65, GAPDH, and Lamin A/C. GAPDH and Lamin A/C were used as loading controls for the cytosolic and nuclear fractions, respectively. Representative images are shown (*n* *=* 5; *P *<* 0.05, static vs. LSS or OSS; ^&^*P* < 0.05, LSS vs. OSS; ^#^*P* < 0.05, OSS vs. siCAR + OSS). **b** The knockdown of CAR by siCAR was validated by western blotting. Levels of mRNAs encoding anti-inflammatory genes (eNOS), proinflammatory genes (VCAM-1 and ICAM-1), and GAPDH (internal control) were measured by RT-PCR. Representative images are shown (*n* = 5; *P < 0.05, static vs. LSS or OSS; ^#^*P* < 0.05, OSS vs. siCAR + OSS). **c** The protein levels of eNOS, VCAM-1, and ICAM-1 were measured by western blotting. Representative images are shown (*n* *=* 5; *P *<* 0.05, static vs. LSS or OSS; ^&^*P* < 0.05, LSS vs. OSS; ^#^*P* < 0.05, OSS vs. siCAR + OSS). **d** To assay monocyte binding to endothelial cells, we exposed transfected HUVECs to FSS for 24 h. Monocytes in five random optical fields per sample were counted (*n* = 5; *P < 0.05, static vs. LSS or OSS; ^&^*P* < 0.05, LSS vs. OSS; ^#^*P* < 0.05, OSS vs. siCAR + OSS).

Disturbed flow induces endothelial inflammation via activation of the NF-κB pathway. Activation of NF-κB is mediated by phosphorylation of the inhibitory subunit, IκB, and nuclear translocation of NF-κB p65. To explore the role of CAR in ECs, we determined the effects of CAR on disturbed flow-induced NF-κB activation. The phosphorylation of IκB and nuclear translocation of NF-κB p65 were increased in OSS, but this increase was inhibited by CAR knockdown (Fig. 3a). Next, we explored the effects of CAR on downstream signaling by NF-κB: the expression of the anti-inflammatory eNOS gene and the proinflammatory genes, such as the vascular cell adhesion molecule 1 (VCAM-1) and intercellular adhesion molecule 1 (ICAM-1) in CAR knockdown HUVECs. eNOS was significantly downregulated at the mRNA (Fig. 3b) and protein (Fig. 3c) levels, whereas VCAM-1 and ICAM-1 were upregulated under OSS compared with LSS. Interestingly, the OSS-mediated reduction in eNOS expression was increased by CAR knockdown, whereas the increased levels of VCAM-1 and ICAM-1 under OSS were suppressed by CAR knockdown. To assess the functional relevance of the CAR knockdown-mediated reduction in the levels of OSS-induced adhesion molecules, we evaluated the adhesion of monocytes to HUVECs. CAR knockdown inhibited the OSS-induced increase in monocyte adhesion to ECs (Fig. 3d).

### CAR deletion has protective effects in the proatherogenic endothelium under disturbed flow

**Fig. 4:**
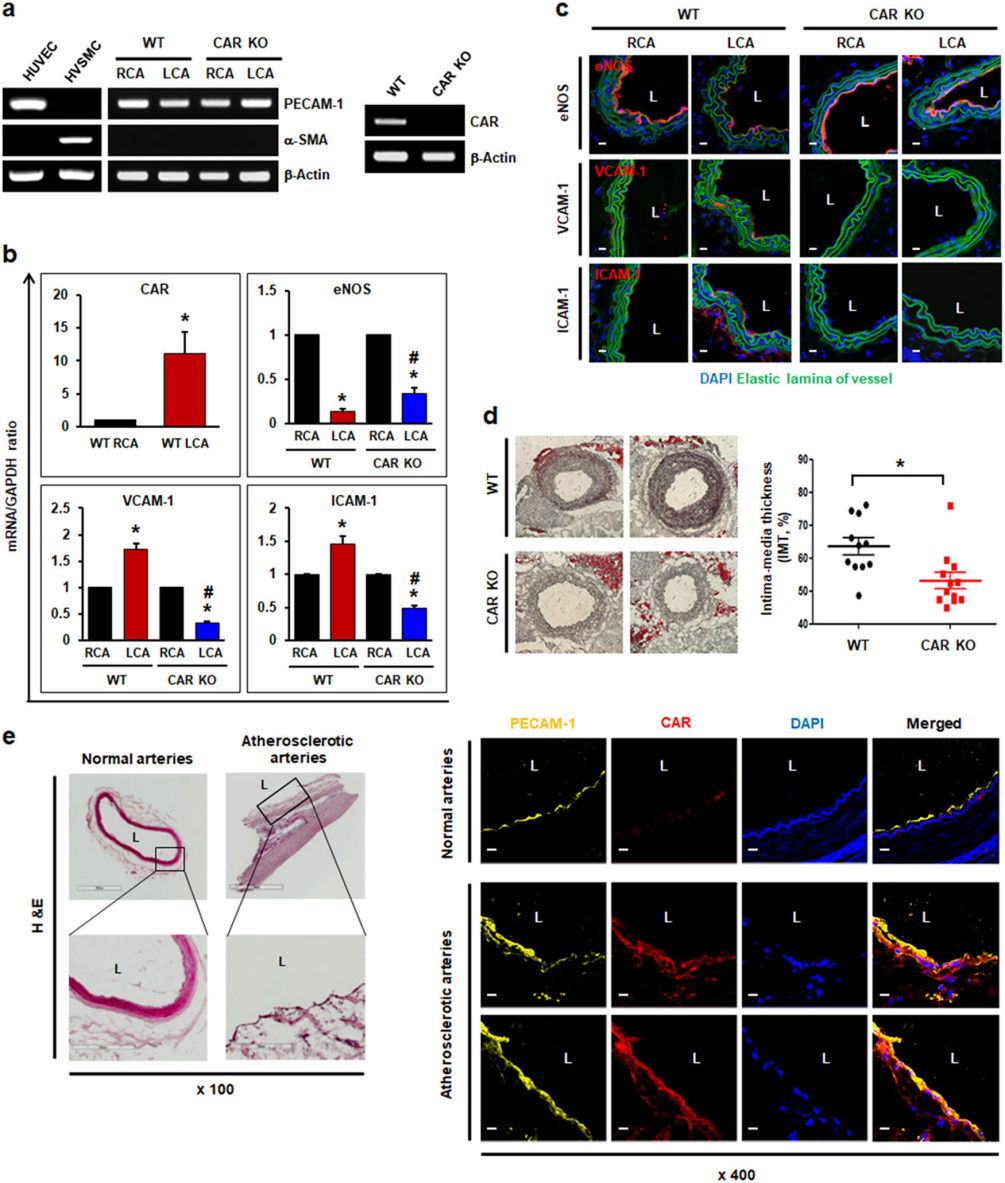
CAR deletion protects the proatherogenic endothelium under disturbed flow. Wild-type (WT) C57BL/6 or EC-specific CAR KO mice were partially ligated in the LCA. **a** Twenty-four hours after LCA ligation, the mice were euthanized, and EC-enriched RNA was extracted from both carotid arteries. EC-enriched RNA was confirmed by assessing an endothelial-specific marker (PECAM-1), a smooth muscle cell-specific marker (α-smooth muscle actin [α-SMA]), and β-actin (internal control). HUVECs were used as a positive control for PECAM-1 and a negative control for α-SMA. Human vascular smooth muscle cells (VSMCs) were used as a positive control for α-SMA and a negative control for PECAM-1. The knockout of endothelial CAR in EC-specific CAR KO mice was confirmed by RT-PCR. Representative images are shown (*n* = 5). **b** Levels of mRNAs encoding eNOS, VCAM-1, and ICAM-1 in EC-enriched RNA were determined by real-time PCR (*n* = 5; *P < 0.05, WT RCA vs. WT LCA, CAR KO RCA vs. CAR KO LCA; ^#^*P* < 0.05, WT LCA vs. CAR KO LCA). **c** Immunofluorescence staining was performed in carotid arteries from WT or EC-specific CAR KO mice. Representative images are shown (*n* = 3; red, eNOS, VCAM-1, or ICAM-1; blue, nuclei; green, elastic lamina of vessel; scale bars, 10 μm). **d** Disturbed flow-induced atherosclerotic plaque formation was visualized in ligated LCA by Oil Red O staining. We measured the IMT by calculating the luminal area relative to the total vascular area. Representative images are shown (*n* = 11; *P < 0.05, WT vs. CAR KO). **e** Human arterial tissues were stained with hematoxylin and eosin (H&E) and anti-PECAM-1 and anti-CAR antibodies. PECAM-1 was used as an endothelial-specific marker. Expression of CAR was compared between normal and atherosclerotic regions of arterial tissue by confocal microscopy. Representative images from at least three experiments are shown (red, CAR; yellow, PECAM-1; blue, nuclei). Scale bars, 10 μm.

Next, we determined the effects of CAR deletion on the endothelium in vivo by generating EC-specific CAR KO mice on a C57BL6 background and utilizing partial carotid artery ligation as a model of disturbed flow-induced atherosclerosis^19,20^. After 3 days of partial carotid artery ligation, the expression of anti- and proinflammatory genes in wild-type (WT) and EC-specific CAR KO mice was evaluated. The total RNA was isolated from the endothelia of carotid arteries, and the presence of an endothelial-specific marker (PECAM-1) was confirmed, whereas a smooth muscle cell-specific marker (α-smooth muscle actin [α-SMA]) was absent, as determined by RT-PCR. RT-PCR confirmed efficient CAR KO in ECs (Fig. 4a). In the WT mice, CAR expression was upregulated in the ligated left carotid artery (LCA) with disturbed flow compared with the unligated right carotid artery (RCA) with normal laminar flow, which is consistent with the results of in vitro experiments. Similarly, the level of the anti-inflammatory marker eNOS was significantly reduced, whereas levels of the proinflammatory markers VCAM-1 and ICAM-1 were increased, in the ligated LCA, as assessed by real-time PCR and immunohistochemistry. However, in EC-specific CAR KO mice with disturbed flow, this increase in the levels of VCAM-1 and ICAM-1 was not observed. The reduction in eNOS expression was less than that observed in WT mice with a ligated LCA (Fig. 4b, c). Four weeks after partial carotid artery ligation and consumption of a western diet, intima-media thickness (IMT), a surrogate marker for atherosclerosis in carotid arteries, was increased in the WT mice. In contrast, EC-specific CAR KO mice exhibited a decreased IMT compared with WT mice (Fig. 4d). In addition, we compared endothelial CAR expression in normal and atherosclerotic regions of human arterial tissue. As expected, regions of atherosclerotic plaque showed higher levels of CAR expression than normal arterial regions (Fig. 4e). Taken together, our results reveal that CAR depletion exerts antiatherogenic effects by blocking the inflammatory response in inflamed ECs exposed to disturbed flow, and that CAR plays an important role in the increased IMT observed following carotid artery ligation.

### CAR modulates FSS-induced endothelial mechanotransduction by regulating PECAM-1 phosphorylation

**Fig. 5:**
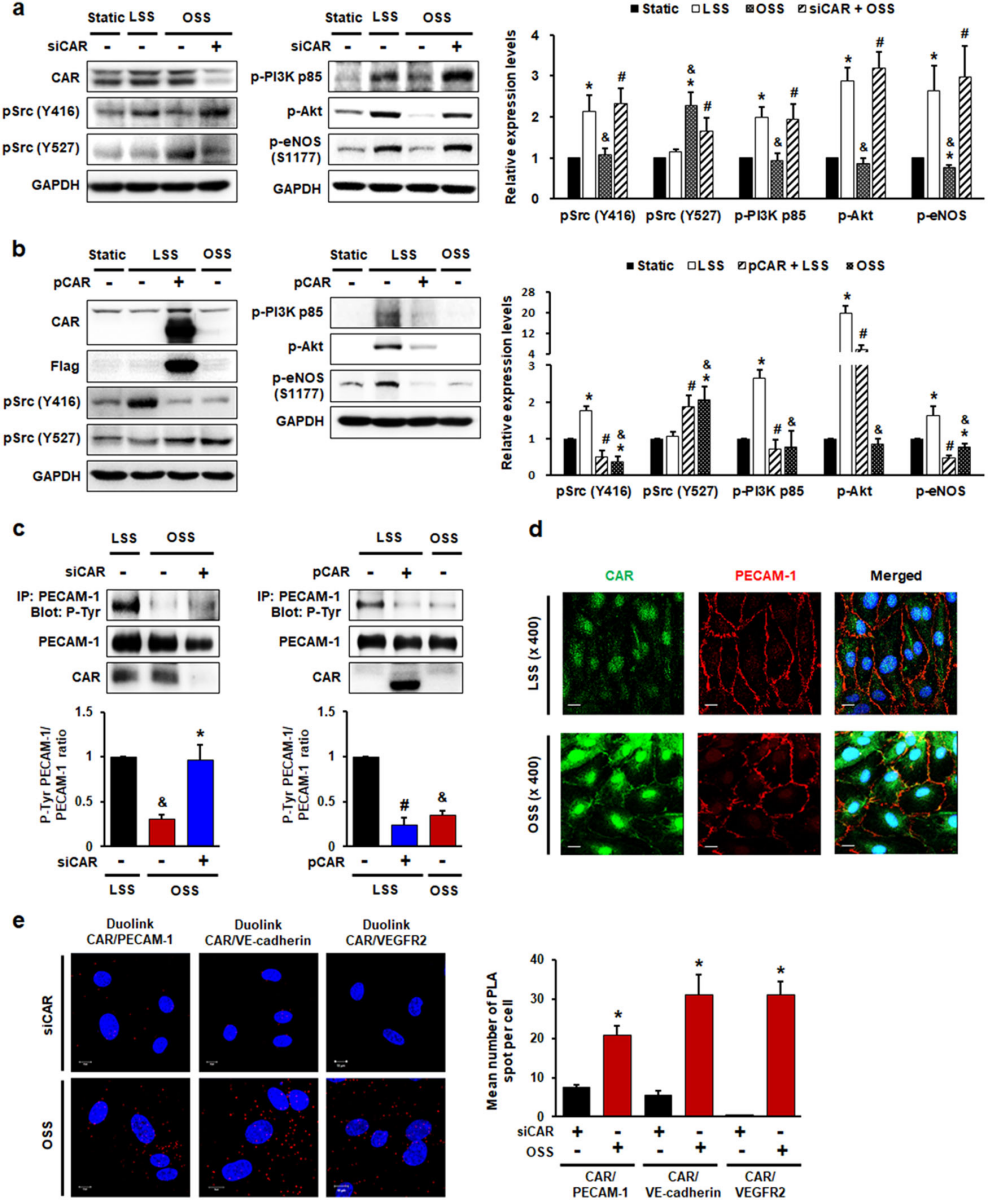
CAR acts as a mechanotransduction modulator in endothelial cells under disturbed flow. HUVECs transfected with siRNA (**a**, **c**, **e**) against CAR or flag-tagged plasmid (**b**, **c**) encoding CAR were exposed to FSS. The phosphorylation of Src at Y416 (active form) and Y527 (inhibitory form), PI3K, Akt, and eNOS (S1177) was assessed. Representative images from at least five experiments are shown. **a** *n* = 5; *P < 0.05, static vs. LSS or OSS; ^&^*P* < 0.05, LSS vs. OSS; ^#^*P* < 0.05, OSS vs. siCAR + OSS. **b** *n* = 5; *P < 0.05, static vs. LSS or OSS; ^&^*P* < 0.05, LSS vs. OSS; ^#^*P* < 0.05, LSS vs. pCAR + LSS. **c** Transfected HUVECs exposed to FSS for 10 min were immunoprecipitated with anti-PECAM-1 antibody. The tyrosine phosphorylation of PECAM-1 was assessed in PECAM-1 immunoprecipitates by western blotting (*n* = 5; ^&^*P* < 0.05, LSS vs. OSS; *P < 0.05, OSS vs. siCAR + OSS; ^#^*P* < 0.05, LSS vs. pCAR + LSS). **d** Colocalization of CAR with PECAM-1 was visualized by confocal microscopy. Representative images from at least three experiments are shown (green, CAR; red, PECAM-1; blue, nuclei; scale bars, 10 μm). **e** The association between CAR and the mechanosensory complex (VEGFR2, VE-cadherin, and PECAM-1) in HUVECs exposed to disturbed flow was determined by PLA. Cells transfected with siCAR were used as a negative control. Red spots indicate interactions between CAR and the mechanosensory complex. Red spots were enumerated to quantify the number of in situ PLA signals per cell. Representative images are shown (*n* *=* 5; compared with siCAR, *P *<* 0.05; scale bars, 10 μm).

Shear stress induces the rapid tyrosine phosphorylation of mechanosensors and activates Src family kinases. The activation of Src results in the phosphorylation of PI3K, Akt, and eNOS in ECs^3,29–31^. To explore the functions of CAR in mechanotransduction signaling in the response of ECs to shear stress, we evaluated the phosphorylation of Src, PI3K, Akt, and eNOS in HUVECs transfected with siCAR or a plasmid-encoding CAR. LSS induced the activation of Src (pY416, active form), PI3K, Akt, and eNOS, whereas OSS did not activate these molecules. In terms of Src phosphorylation, the Y527 inhibitory form of Src was increased in OSS compared with LSS. Reduced phosphorylation of Src (Y416), PI3K, Akt, and eNOS in OSS was reversed by CAR knockdown, whereas phosphorylation of Src (Y527) was suppressed (Fig. 5a). In contrast, the activation of Src (Y416), PI3K, Akt, and eNOS and reduced Src phosphorylation (Y527) induced by LSS was abolished by CAR overexpression (Fig. 5b).

A mechanosensory complex (composed of PECAM-1, VE-cadherin, and VEGFR2) mediates the endothelial mechanotransduction response to FSS^32,33^. PECAM-1 may be a key molecule in this complex^3,34^. The PECAM-1 cytoplasmic domain can directly bind Src, and is required for Src activation^35^. Phosphorylation of the PECAM-1 cytoplasmic domain regulates the assembly of signaling complexes and interactions with various elements of the cytoskeleton^36^. To determine the effects of CAR on PECAM-1 phosphorylation, we evaluated tyrosine phosphorylation in PECAM-1 immunoprecipitates. The reduced phosphorylation of PECAM-1 under OSS was recovered by CAR knockdown, whereas enhanced PECAM-1 phosphorylation under LSS was abolished by CAR overexpression (Fig. 5c). Using confocal microscopy, we found that CAR colocalized with PECAM-1 (Fig. 5d). Therefore, we investigated endogenous protein–protein interactions between CAR and the mechanosensory complex in HUVECs exposed to disturbed flow by immunostaining-based in situ proximity ligation assay (PLA). PLA revealed that CAR, the expression of which was increased under OSS, was physically associated with the mechanosensory complex in HUVECs (Fig. 5e). Taken together, these findings show that CAR has a close physical relationship with PECAM-1 and other mechanosensory complex proteins in ECs, and that PECAM-1 phosphorylation is regulated by CAR through the interaction between CAR and the mechanosensory complex or other proteins under FSS conditions. These observations suggest that CAR is involved in the earliest signaling step in mechanotransduction by ECs under FSS through the regulation of PECAM-1 phosphorylation. In addition, FSS induces changes in focal adhesion and cytoskeletal organization, followed by stress fiber formation in ECs. Since CAR was found to influence endothelial mechanotransduction signaling under FSS, we further explored the effect of CAR on stress fiber formation in the direction of flow. As a result, LSS induced the elongation of ECs and directional stress fiber organization along the direction of flow, but cells under static or OSS conditions showed a cobblestone-like shape and exhibited loosely arranged stress fibers. However, CAR knockdown had no effect on the response of endothelial stress fiber formation to FSS (Supplementary Fig. 5).

## Discussion

**Fig. 6:**
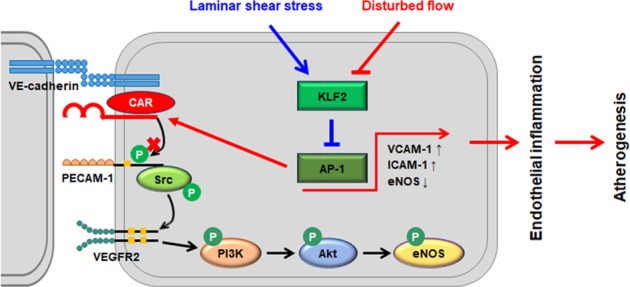
Schematic of the proposed role of CAR in endothelial mechanotransduction signaling under FSS. Endothelial CAR expression is regulated by FSS. Disturbed flow increases, whereas laminar shear stress decreases, CAR expression in ECs; these changes in CAR expression are regulated through the KLF2-AP-1 axis. Disturbed flow-induced CAR inhibits the phosphorylation of PECAM-1, leading to the suppression of endothelial mechanotransduction signaling through the phosphorylation of Src, PI3K, Akt, and eNOS. Therefore, CAR modulates mechanotransduction in ECs by blocking PECAM-1 phosphorylation, which influences endothelial function and vascular pathophysiology.

The major finding of this study is that CAR, a viral receptor, is regulated by vascular FSS through the KLF2/AP-1 axis in ECs. The deletion of CAR downregulated the expression of proinflammatory genes, reducing endothelial inflammation under disturbed flow both in vitro and in vivo, followed by delayed atherogenesis. Furthermore, CAR is involved in the mechanosensing pathway through its interaction with the mechanosensory complex and regulation of PECAM-1 phosphorylation (Fig. 6). To the best of our knowledge, this is the first study of the expression and role of CAR in ECs exposed to FSS conditions.

Vascular ECs are directly exposed to various blood flow patterns, which modulate endothelial function and vascular pathophysiology. CAR has recently attracted much attention as a tumor suppressor in addition to its role as a primary receptor for adenoviral vectors used for cancer gene therapy. Moreover, CAR plays a role in cardiomyopathy. However, the role of CAR in ECs has not yet been elucidated. Based on both in vitro and in vivo studies, we found that changes in the blood flow pattern affected CAR expression; disturbed flow upregulated, whereas LSS downregulated, CAR expression at both the mRNA and protein levels. FSS also stimulated shedding of the CAR ectodomain, and OSS conditions induced more shedding of the CAR ectodomain than LSS conditions. Therefore, the regulation of endothelial CAR by FSS is not due to shedding of the CAR ectodomain.

We determined the mechanism underlying the regulation of endothelial CAR expression by FSS. Little is known about the regulatory mechanism of CAR expression in ECs; most studies have been limited to tumor cells. In cancer cells, the Raf/MEK/ERK pathway^37^ and the transcription factor Sp1 regulate CAR expression. In addition, the histone deacetylase (HDAC) inhibitors FR901228^38^ and trichostatin A (TSA)^39^ increase CAR mRNA levels through epigenetic chromatin remodeling events, such as histone acetylation. However, the regulatory mechanism of CAR expression in ECs constantly exposed to FSS is unclear. Because KLF2 is a key shear stress-induced transcription factor that governs the expression of many flow-regulated endothelial genes^26^, we focused on KLF2 as a regulator of CAR expression. We determined the effect of KLF2 knockdown or overexpression on CAR expression in ECs. Decreased CAR expression under LSS was increased by KLF2 knockdown, whereas increased CAR expression under OSS was decreased by KLF2 overexpression. This finding implies that KLF2 is associated with endothelial CAR expression under flow conditions. KLF2 reportedly downregulates the expression of proinflammatory genes by inhibiting AP-1^27,40^ and NF-κB activation^28^. Interestingly, our results show that the inhibition of AP-1 resulted in a reduction in disturbed flow-induced expression of CAR, but not NF-κB. Next, we examined whether the suppression of CAR by KLF2 is mediated by AP-1 activation blockade. KLF2 overexpression suppressed OSS-induced AP-1 activation, whereas KLF2 knockdown increased AP-1 activation compared with that under LSS conditions. Therefore, the regulation of endothelial CAR expression by FSS is mediated by the flow-responsive KLF2/AP-1 axis.

In carcinomas, CAR regulates cancer cell adhesion, proliferation, migration, and invasion. Furthermore, the loss of CAR promotes cancer development^8–11^. However, little is known about the role of CAR in ECs. CAR mediates the transendothelial migration of neutrophils^14^. To explore the role of CAR in ECs, we examined the effects of CAR knockdown on NF-κB activity and its downstream signaling; the expression of anti- and proinflammatory genes. Disturbed flow-induced IκB phosphorylation and the nuclear translocation of NF-κB p65 were suppressed by CAR knockdown. Therefore, CAR knockdown reduced endothelial inflammation and the expression of proinflammatory genes that are increased under disturbed flow via suppressing NF-κB activation. Consistent with the in vitro results, these results show that disturbed flow-induced atherosclerosis was reduced in EC-specific CAR KO mice. In human arterial tissues, the atherosclerotic regions showed higher levels of endothelial CAR expression than normal regions. Therefore, our results suggest that CAR plays an important role in the development of disturbed flow-induced atherosclerosis.

Because CAR knockdown abolished the effects of disturbed flow on ECs, we investigated the role of CAR in endothelial mechanotransduction signaling induced by shear stress. Mechanosensors composed of various cellular components—such as integrins, caveolae, ion channels, the glycocalyx, and G-proteins—allow cells to respond to shear stress and activate mechanotransduction signaling^41–45^. FSS induces rapid tyrosine phosphorylation of mechanosensors and activates Src family kinases. The activation of Src leads to the phosphorylation of PI3K, Akt, and eNOS in ECs^3,29–31^. A Src family kinase is located upstream of PI3K, as PI3K phosphorylation was prevented by the Src kinase inhibitor PP2^3^, and PECAM-1 directly transmits mechanical force and is required for Src activation. Therefore, we examined the effects of CAR on the phosphorylation of PECAM-1, an upstream signaling molecule in LSS-induced mechanotransduction. The decreased phosphorylation of PECAM-1 under OSS was recovered by CAR knockdown, whereas the enhanced PECAM-1 phosphorylation under LSS was abolished by CAR overexpression. Moreover, CAR had a close physical relationship with PECAM-1 and other mechanosensory complex proteins in ECs, as determined by immunostaining-based PLA. These results suggest that PECAM-1 phosphorylation is regulated by CAR through its interaction with the mechanosensory complex or other proteins under FSS conditions. Consequently, CAR is involved in the earliest signaling step in FSS-induced mechanotransduction through its regulation of PECAM-1 phosphorylation in ECs. However, CAR had no effects on FSS-induced stress fiber formation, even though CAR was found to regulate endothelial mechanotransduction signaling by FSS.

In summary, this study provides novel evidence that FSS regulates CAR expression in ECs and that disturbed flow upregulates CAR through the KLF2/AP-1 pathway. Inhibition of CAR expression downregulates the expression of proinflammatory genes via the suppression of NF-κB activation, thereby reducing endothelial inflammation under disturbed flow both in vitro and in vivo, followed by delayed atherogenesis. CAR inhibits the phosphorylation of PECAM-1, leading to the suppression of endothelial mechanotransduction signaling. These results indicate that CAR is an important modulator of mechanotransduction in ECs that acts by blocking PECAM-1 phosphorylation, which influences endothelial function and vascular pathophysiology. Therefore, CAR is involved in the maintenance of vascular function and shows promise as a target for the development of therapies for disturbed flow-induced vascular diseases, such as atherosclerosis.

## Supplementary information

Supplemental material
