# Supplementary material for: Coxsackievirus and adenovirus receptor mediates the responses of endothelial cells to fluid shear stress
Source: Exp Mol Med. 2019 Nov 27;51(11):144. doi: 10.1038/s12276-019-0347-7 (PMC6881322; doi:10.1038/s12276-019-0347-7)
Supplement: Supplementary file 1 — Supplemental material [file 12276_2019_347_MOESM1_ESM.doc]

**Coxsackievirus and adenovirus receptor mediates the responses of endothelial cells to fluid shear stress**

Jihwa Chung^1^, Kyoung Hwa Kim^1^, Shung Hyun An^1^, Sunmi Lee^3^, Byung Kwan Lim^4^, Sang Won Kang^3^, Kihwan Kwon* ^1,2^

**
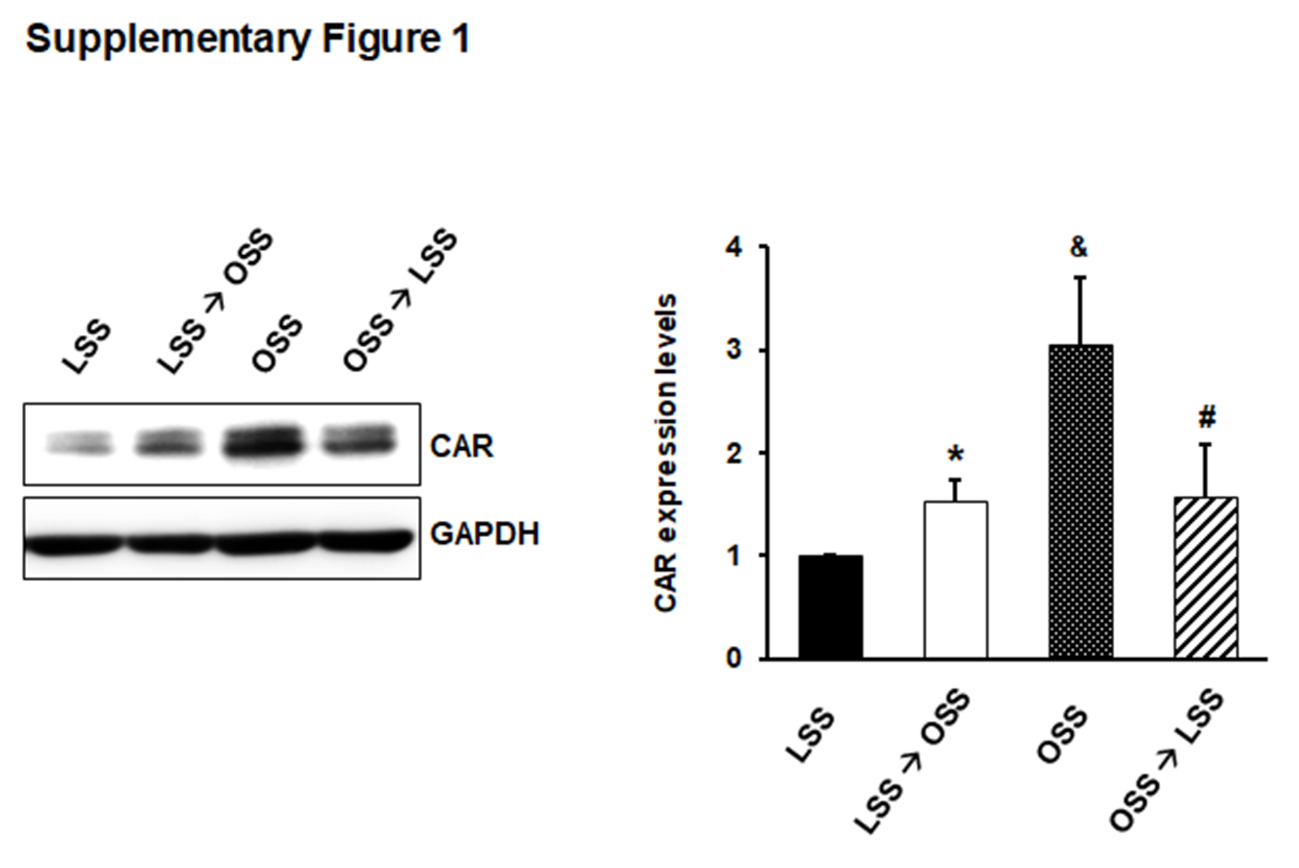
**

**Supplementary figure 1. Disturbed flow upregulates CAR expression in endothelial cells.**

After FSS stimulation for 24 h HUVECs were crossed over between LSS and OSS. CAR protein levels were measured by Western blotting. Representative images are shown (*n* = 7; **P* < 0.05, LSS vs. LSS + OSS; &*P* < 0.05, LSS vs. OSS; #*P* < 0.05, OSS vs. OSS + LSS).

**
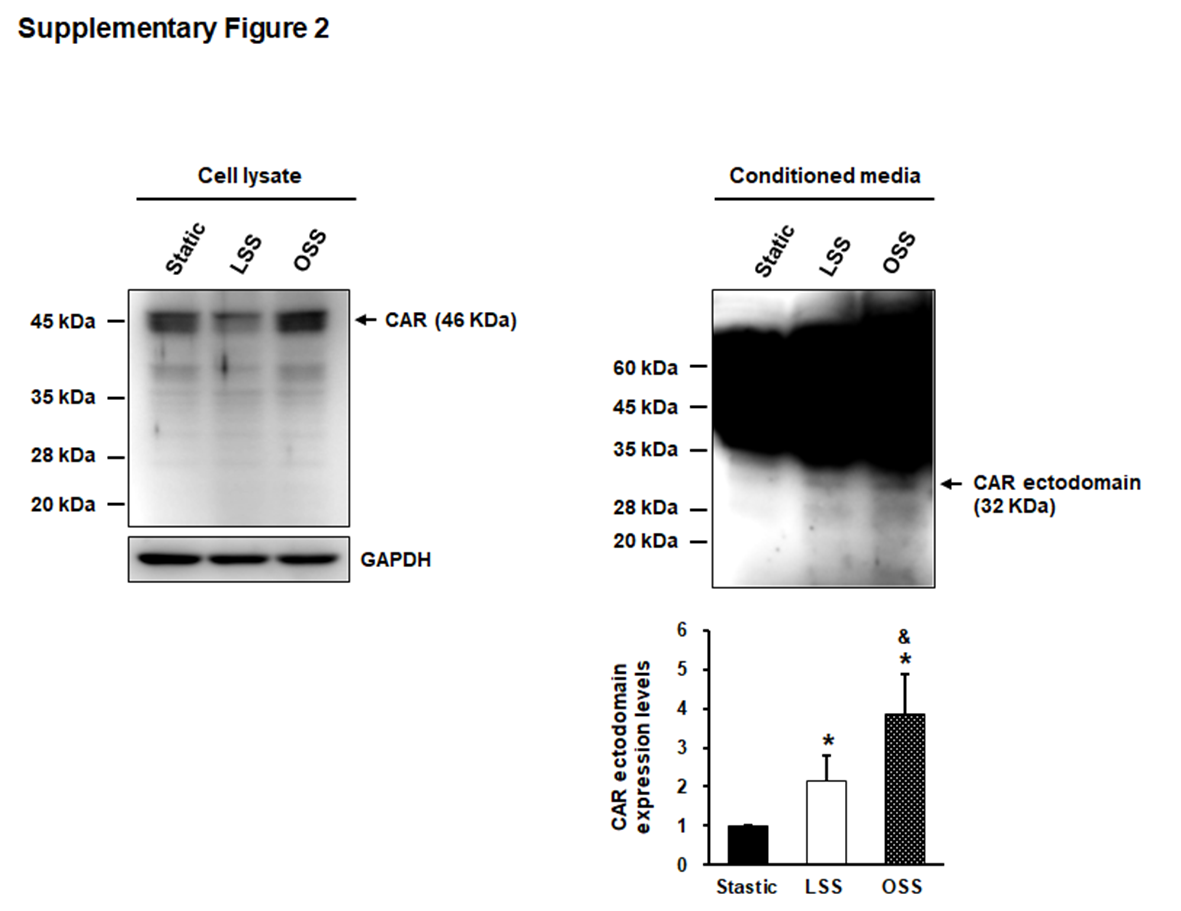
**

**Supplementary Figure 2. FSS induces shedding of CAR extracellular domain (CAR ectodomain).**

Cell lysates and conditioned media were collected from HUVECs exposed to LSS or OSS for 24 h. A Western blotting of conditioned media and cell lysates was performed using the anti-CAR antibody. The full-length CAR detected from cell lysates was at approximately 46 kDa, while CAR ectodomain detected from conditioned media was at approximately 32 kDa. Representative images are shown (*n* = 5; **P* < 0.05, static vs. LSS or OSS; &*P* < 0.05, LSS vs. OSS).


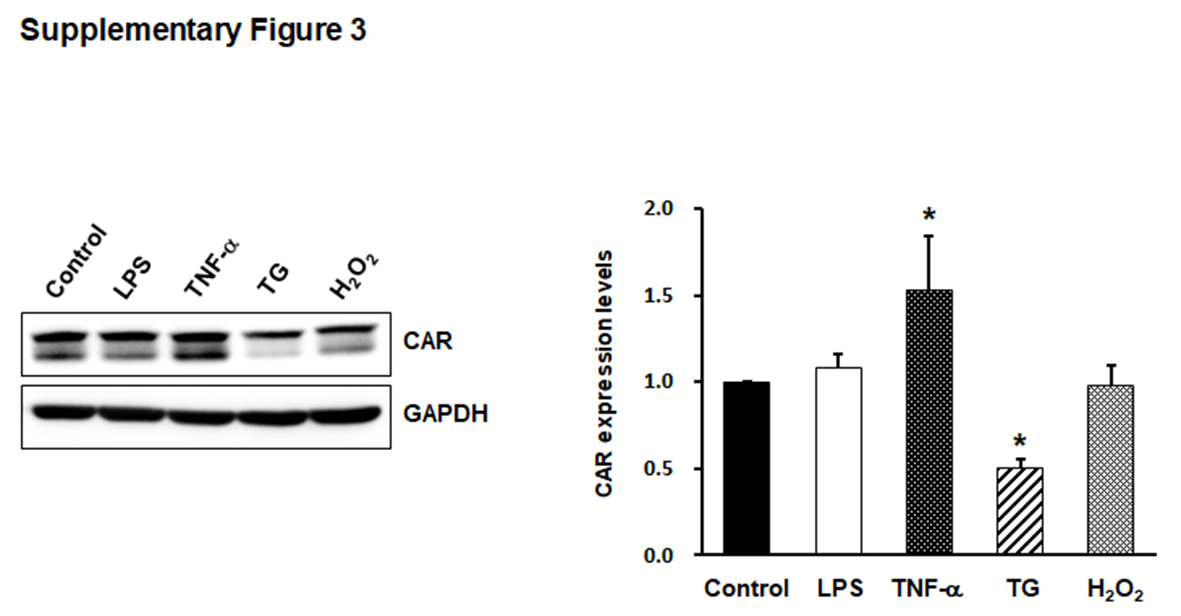


**Supplementary Figure 3. TNF-α upregulate CAR expression in endothelial cells.**

HUVECs were treated with various stimuli such as LPS, TNF-α, thapsigargin (TG), and H_2_O_2_ for 24 h. The protein levels of CAR were measured by Western blotting. Representative images from at least three experiments are shown (*n* = 5; compared to static conditions, **P* < 0.05).


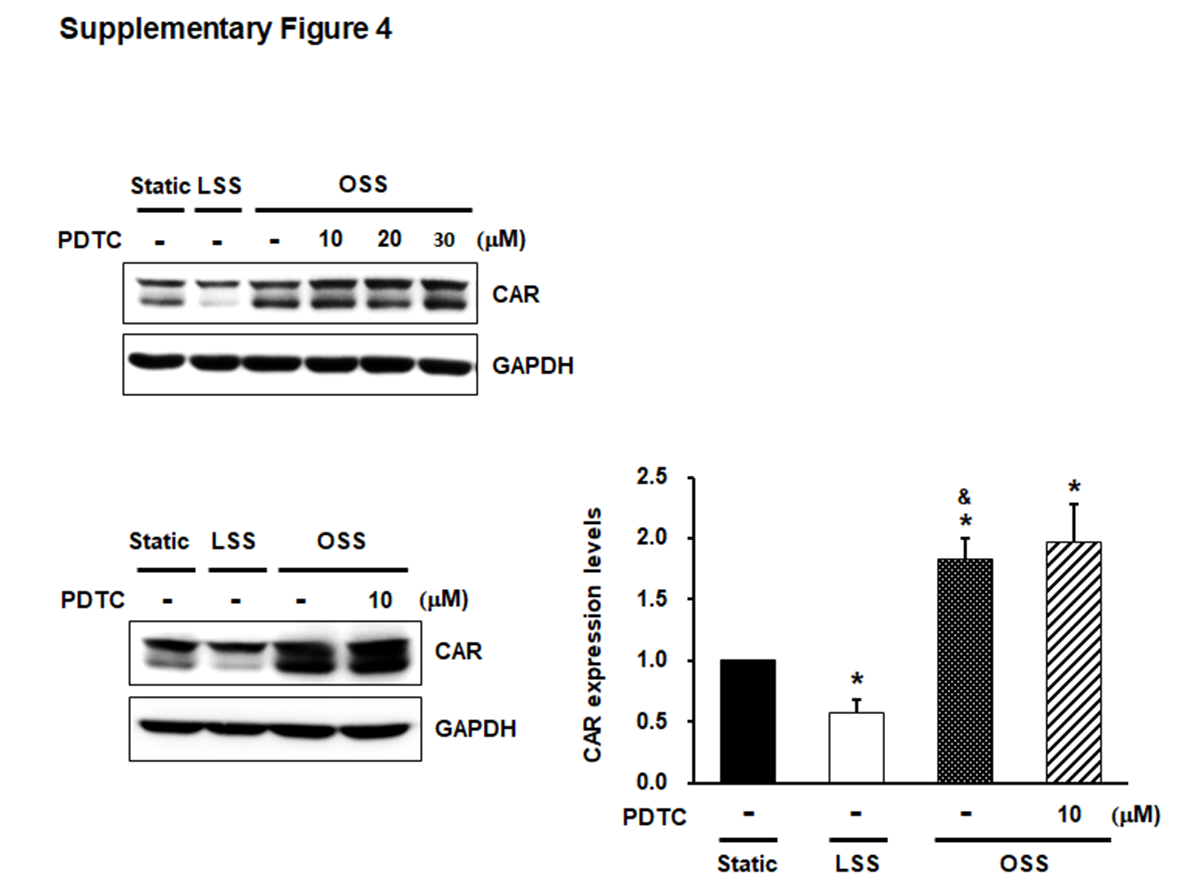


**Supplementary Figure 4. Disturbed flow-induced CAR expression is not involved in NF-κB activation in endothelial cells.**

HUVECs were pretreated with various doses of the NF-κB inhibitor PDTC for 1 h and exposed to LSS or OSS for 24 h. CAR protein levels were measured by Western blotting. Representative images are shown (*n* = 5; **P* < 0.05, static vs. LSS or OSS; &*P* < 0.05, LSS vs. OSS).


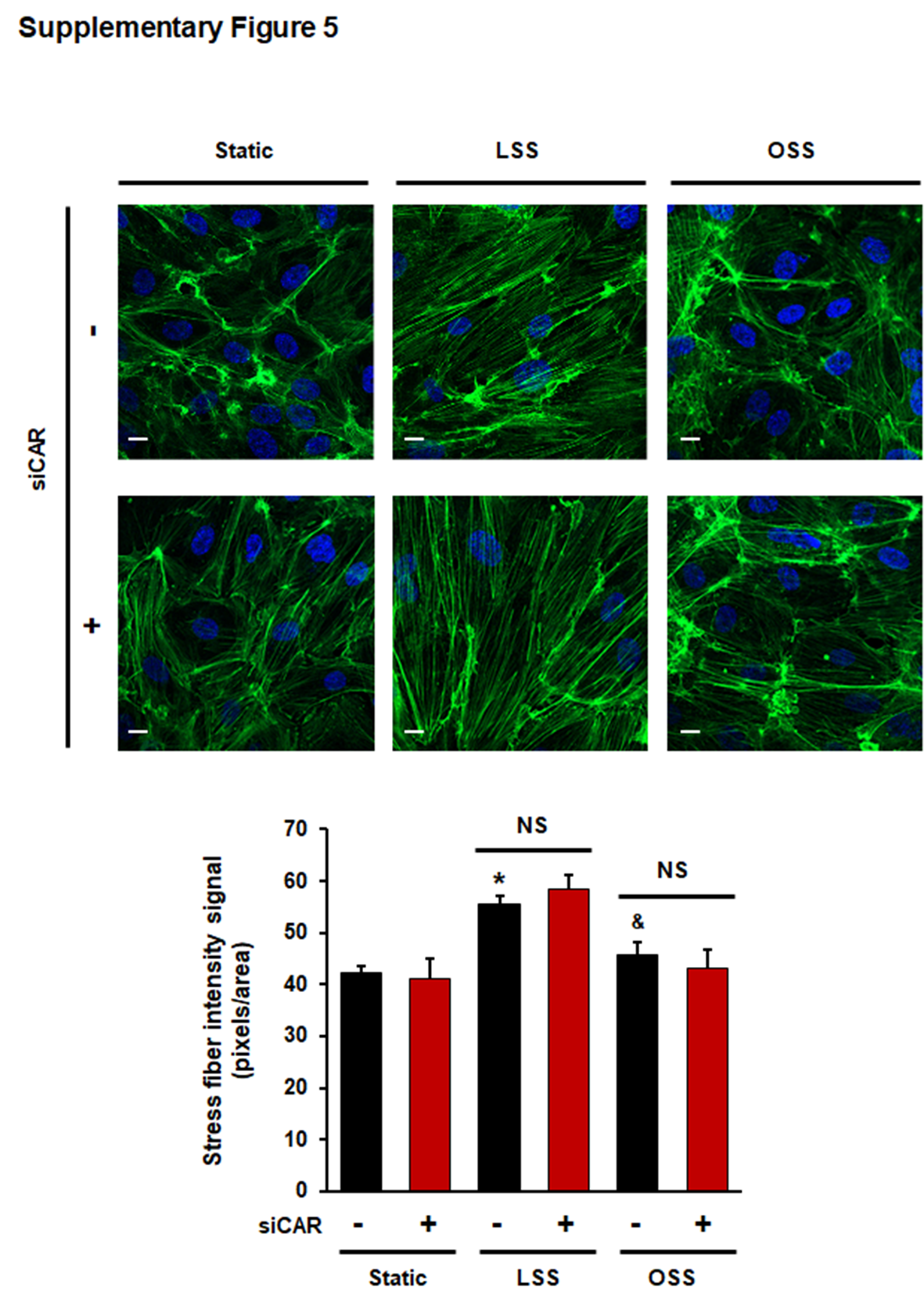


**Supplementary Figure 5. Depletion of CAR has no effects on the stress fiber formation in response to shear stress in endothelial cells.**

HUVECs transfected with siRNA against CAR were exposed to FSS for 24 h. Cells were fixed and stained with phalloidin conjugate for F-actin. Representative images are shown (green, F-actin; blue, nuclei; scale bars, 10 μm). Stress fiber area was measured and analyzed for signal intensity using ImageJ (*n* = 5; **P* < 0.05, static vs. LSS or OSS; &*P* < 0.05, LSS vs. OSS). NS = no significant.
